# Adverse childhood experiences and their relationship to gender and depression among young adults in Iraq: a cross-sectional study

**DOI:** 10.1186/s12889-019-7957-9

**Published:** 2019-12-16

**Authors:** Ameel F. Al Shawi, Yassen T. Sarhan, Mahasin A. Altaha

**Affiliations:** 1 Department of Community & Family Medicine, College of Medicine, University of Fallujah, Fallujah, Iraq; 2 Department of Community & Family Medicine, College of Medicine, Anbar University, Ramadi, Iraq

**Keywords:** Adverse childhood experiences, Gender, Depression, Young adults, Iraq

## Abstract

**Background:**

Adverse childhood experiences (ACEs) are considered as universal public health problem that associate with mental disorders and risky behaviors during adulthood. The aims of the paper are to estimate the prevalence of Adverse childhood experiences (ACEs) among young adults in Iraq as well as to estimate the association between ACEs and depression.

**Methods:**

A convenience sample of young adults of 18–20 years was chosen from centre and west of Iraq, mainly from universities. The adverse childhood experiences were measured by Adverse Childhood Experiences Questionnaire. A depression scale was derived from the Depression, Anxiety and Stress Scales (DASS). Statistical tests: chi square was used to measure the association between adverse childhood experiences and other variables like gender and depression. Odds ratios were computed to estimate the risk for depression. A *P* value of less than 0.05 was considered statistically significant.

**Results:**

The total number of participants was 401, 38.9% of the subjects were men, while 61.1% were women. The mean age of the participants was 18.88 ± 0.745. The results revealed that the most common forms of ACEs among the subjects were physical neglect (19.8%) and emotional neglect (19.2%) followed by physical abuse (17.21%) while sexual abuse was 7.52%.

There was statistically significant association between most forms of adverse childhood experiences especially emotional abuse, emotional neglect, physical abuse and physical neglect with depression.

**Conclusion:**

ACEs are not uncommon among young adults in Iraq and are associated with depression in adulthood. National programmes to support mental health rehabilitation might be necessary to reduce the effect of ACEs among Iraqi people, especially for adolescents and young adults.

## Background

Adverse Childhood Experiences (ACEs) can be defined as ‘all types of abuse, neglect, and other potentially traumatic experiences that occur to people under the age of 18’ [1].

The literature has documented that ACEs are a global and universal issue that can have complicated and serious effects on physical, mental and social health during adulthood, also can lead to risky behaviours like alcohol consumption, drug abuse and even suicide attempts [2–5].

The association between exposure to traumatic events during childhood and the development of depression during adulthood has been addressed by several studies, which have demonstrated that the exposure of children to different kinds of violence leads to emotional, neurological deficits and maldevelopment that may occur not only during childhood but also later in life [6–9].

The prevalence of ACEs varies across the world, and while many studies reported a high prevalence of ACEs in regions with low per capita incomes, there are few longitudinal studies that address the effect of ACEs [9, 10].

In Iraq, studies that reported the prevalence of ACEs and their effects on adults, especially young adults, are scarce. This is despite the fact that Iraqi population have been exposed to different types of violence during recent decades, especially after 2003, that have had a negative impact on the mental health and behaviours of individuals and families [11, 12]. Only one previous study was carried out in Baghdad city in 2015 to estimate ACEs among Iraqis [11].

The current study draws on the Iraqi sample from the ‘International study of pro/antisocial behavior in young adults (SOCIALDEVIANCE1820)’ which is an ongoing cross-continental longitudinal study that includes data and researchers from multiple countries. Its main objective is to measure the universality of ACEs as risk factors for psychosocial problems [13].

The aims of this paper are to estimate the prevalence of ACEs among young adults in Iraq and to estimate the association between ACEs and depression.

## Methods

### Study design

A cross-sectional study was conducted during the period from October 2018 to April 2019.

### Study subjects

A convenience (non - probability) sample of young adults of 18–20 years was chosen from the centre and west of Iraq, mainly from universities (Baghdad, Al Mustansyria, Al Naharain, Anbar and Fallujah). These universities had students from different governorates in Iraq.

The study includes young adults living in the local community between the ages of 18 and 20 who could provide informed consent. Subjects having less than 4 years of schooling were excluded.

### Questionnaire

The questionnaire consisted of questions for sociodemographic characteristics including age, gender, occupation (work, study, work and study, and do not work or study) and financial issues (Are you financially independent? Yes, No).

ACEs were measured by the ACE questionnaire, which consists of 36 questions and was designed to measure the occurrence of a set of experiences during childhood and adolescence [14]. Such experiences include multiple types of abuse and neglect (physical, sexual and emotional) or household dysfunction (mental illness of a household member, substance abuse by a family member, parental divorce or incarcerated family members) [15]. These questions included, for example *‘While you were growing up, during your first 18 years of life: was anyone in your household depressed or mentally ill?’* and *‘During the first 18 years of your life how often did a parent, guardian or household member punch, kick or beat you?’* The Cronbach’s alpha for the ACE questionnaire in the current study was 0.77*.*

A depression scale was derived from the Depression, Anxiety and Stress Scales (DASS), which is a widely used screening tool to assess symptoms of depression, anxiety, and stress in community settings. This had a good internal consistency, with a Cronbach’s alpha of 0.88 [16].

The depression subscale consists of seven questions that measure hopelessness, low self-esteem and low positive affect [16, 17]. Each item of the depression subscale comprises a statement such as in the past week: *‘I couldn’t seem to experience any positive feelings at all’*, *‘I found it difficult to work up the initiative to do things’*, *‘I felt that I had nothing to look forward to’*, *‘I felt down-hearted and blue’*, *‘I was unable to become enthusiastic about anything’*, *‘I felt I wasn’t worth much as a person’* and *‘I felt that life was meaningless’*. Four short-response options reflect the degree of agreement with the statement, scored from 0 (*Did not apply to me at all*) to 3 (*Applied to me very much, or most of the time*). A total score of 9 and below is considered normal, while a score of 10 and above is considered depressed [16, 18]. The Cronbach’s alpha for the depression subscale in this study was 0.80.

The final version of the questionnaire was translated from English to Arabic by an expert from the Community Medicine Department at the University of Fallujah. The approval for the study was made by the Scientific Committee of the Medical College, University of Fallujah.

### Ethical issues

The self- administered questionnaire form was anonymous. Verbal consent was taken from the participants after explaining the aims of the study, assuring them that all information would be kept strictly confidential.

### Data analysis

Data entry followed by descriptive and analytic statistics were carried out using Excel and the Statistical Package for Social Science (SPSS- version 21).

Frequencies and proportions were calculated during the descriptive analysis. Chi-square was used to measure the association between ACEs and other variables like gender and depression. Odds ratios were computed to estimate the risk for depression. A *P*-value of less than 0.05 was considered statistically significant.

## Results

The total number of participants was 401, 38.9% of the subjects were men, while 61.1% were women. The mean age of the participants was 18.8 ± 0.7 years. 6.2% of the sample was financially independent (Table 1).

**Table 1:** Sociodemographic characteristics of the subjects

	n	%
Gender		
Males	156	38.9
Females	245	61.1
Total	401	100.0
Occupation		
Working	3	0.7
Studying	381	95.0
Working and Studying	16	4.0
Did not study or work	1	0.2
Total	401	100.0
Financially Independent		
Yes	25	6.2
No	375	93.5
Total	400*	99.8

*< 401 due to missed questions

Regarding the prevalence of ACEs among the participants, the results showed that the most common forms of ACE were physical neglect (19.8%) and emotional neglect (19.2%), then physical abuse (17.2%). Exposure to domestic violence was reported by 16.5% of the subjects, mental illness in the household was reported by 8.2% of the participants, while the proportion of participants reporting sexual abuse was 7.5%. The ACE score showed that 17.2% of the subjects experienced one ACE, 16.5% of the subjects experienced two ACEs, while 7.5% of the participants experienced four or more ACEs (Table 2).

**Table 2:** Frequency of ACEs among the participants

	Total		Gender				
			Males		Females		
	N	%	n	%	n	%	P*
Emotional Abuse							
no	374	93.3	142	91.6	232	95.1	0.163
yes	25	6.27	13	8.4	12	4.9	
Physical Abuse							
no	332	82.79	117	75	215	87.8	0.001
yes	69	17.21	39	25	30	12.2	
Sexual Abuse							
no	369	92.48	136	87.	233	95.5	0.004
yes	30	7.52	19	12.3	11	4.5	
Emotional Neglect							
no	320	79.8	125	81.7	195	80.8	0.413
yes	76	19.2	28	18.3	48	19.2	
Physical Neglect							
no	316	80.2	123	79.9	193	80.4	0.894
yes	78	19.8	31	20.1	47	19.6	
Exposure to domestic violence							
no	333	83.46	126	80.8	207	85.2	0.247
yes	66	16.54	30	19.2	36	14.8	
Substance Abuse household							
no	388	96.76	152	97.4	236	96.3	0.541
yes	13	3.24	4	2.6	9	3.7	
Mental illness in household							
no	368	91.8	138	88.5	230	93.9	0.054
yes	33	8.23	18	11.5	15	6.1	
ACEs score							
0	193	48.1	64	43	129	55.6	0.125
1	69	17.2	31	20.8	38	16.4	
2	66	16.5	27	18.1	39	16.8	
3	23	5.7	11	7.4	12	5.2	
4+	30	7.5	16	10.6	14	6	

*P for chi square

Regarding gender and ACEs, the most prevalent form of ACEs among men was physical abuse (25%) while the most prevalent form of ACEs among women was physical neglect (19.6%). The rate of sexual abuse among male subjects was 12.3% and among female subjects was 4.5%. Generally the prevalence of abuse forms (physical and emotional) was higher among men than women. More details are provided in Table 2.

The results revealed that there was statistically significant association between most forms of ACE, especially emotional abuse (OR = 2.29, C.I. = 1.01–5.17), emotional neglect (OR = 2.78, C.I. = 1.69–4.64), physical abuse (OR = 1.71, C.I. = 1.00–2.90) and physical neglect (OR = 2.13, C.I. = 1.28–3.53) with depression. There was a statistically non-significant association between exposure to domestic violence and depression. On the other hand, there was a statistically significant association between a high ACE score and depression (Table 3).

**Table 3:** The association between ACEs and depression

ACEs	Depression category				
	Non-depressed	Depressed	P*	*OR*	*95% CI*
	n (%)	n (%)			
Emotional Abuse					
no**	254 (95.5)	120 (90.2)	0.041	2.293	1.01-5.17
yes	12 (4.5)	13 (9.8)			
Emotional Neglect					
no	228 (86.4)	91 (69.5)	0.001	2.784	1.66-4.64
yes	36 (13.6)	40 (30.5)			
Physical Abuse					
no	229 (85.4)	103 (77.4)	0.046	1.71	1.00-2.90
yes	39 (14.5)	30 (22.6)			
Physical Neglect					
no	222 (84.4)	94 (71.8)	0.003	2.131	1.28-3.53
yes	41 (15.6)	37 (28.2)			
Sexual Abuse					
no	251 (94.4)	118 (88.7)	0.044	2.127	1.00-4.49
yes	15 (5.6)	15 (11.3)			
Exposure to domestic violence					
no	224 (84.2)	109 (82)	0.568	1.147	0.67-2.03
yes	42 (15.8)	24 (18)			
Substance Abuse household					
no	262 (97.8)	126 (94.7)	0.107	2.426	0.79-7.36
yes	6 (2.2)	7 (5.3)			
Mental illness in household					
no	253 (94.4)	115 (86.5)	0.006	2.64	1.28-5.42
yes	15 (5.6)	18 (13.5)			
ACEs score					
0	142(46.6)	50 (38.8)	<0.001		
1	48(19.1)	21 (16.3)			
2	36(14.3)	30(23.3)			
3	8(3.2)	15 (11.6)			
4+	17(6.8)	13 (10.1)			

*P for chi square

** (no) is the reference category

Regarding the association between ACEs and depression by gender, for men the experiences of sexual or physical abuse as well as physical neglect had a statistically significant association with depression, while for women there was a significant association between emotional or physical neglects and mental illness in households with depression (Table 4).

**Table 4:** Frequency of ACEs forms and gender with depression development

	Gender					
	Males			Females		
	Non depressed	Depression	P*	Non depressed	Depression	P*
	n (%)	n (%)		n (%)	n (%)	
Emotional Abuse						
no	100 (93.5)	42 (87.5)	.216	154 (96.9)	78 (91.8)	.080
yes	7 (6.5)	6 (12.5)		5 (3.1)	7 (8.2)	
Physical Abuse						
no	86 (79.6)	31 (64.6)	.045^*^	143 (89.4)	72 (84.7)	.289
yes	22 (20.4)	17 (35.4)		17 (10.6)	13 (15.3)	
Sexual Abuse						
no	99 (92.5)	37 (77.1)	.007	152 (95.6)	81 (95.3)	.913
yes	8 (7.5)	11 (22.9)		7 (4.4)	4 (4.7)	
Emotional Neglect						
no	89 (84)	36 (77)	0.277	139 (88)	55 (65.5)	<0.001^*^
yes	17 (16)	11 (23)		19 (12)	29 (34.5)	
Physical Neglect						
no	90 (84.1)	33 (70)	.048^*^	132 (84.6)	61 (72.6)	.026^*^
yes	17 (15.9)	14 (30)		24 (15.4)	23 (27.4)	
Exposure to domestic violence						
no	91 (84.3)	35 (72.9)	.097	133 (84.2)	74 (87.1)	.546
yes	17 (15.7)	13 (27.1)		25 (15.8)	11 (12.9)	
Mental illness in household						
no	98 (90.7)	40 (83.3)	.181	155 (96.9)	75 (88.2)	.007^*^
yes	10 (9.3)	8 (16.7)		5 (3.1)	10 (11.8)	

*P for chi square, and significant at level <0.05

## Discussion

ACEs are considered a public health problem [15, 19]. The current study sought to estimate the prevalence of ACEs (abuse, neglect and household dysfunction) among a convenience sample of young adults in Iraq, and the relationship of ACEs to depression symptoms.

The prevalence of physical abuse and neglect was higher than that of adolescents in Brazil (6.9 and 4.6% respectively) [20], in Finland (0.2 and 0.1% respectively) [21], and also higher than what was reported among university students in Germany (3.9 and 4.6% respectively) [3]. The prevalence of physical neglect was higher than for university students in Turkey (5.7%) [4]. The prevalence of domestic violence was higher than that reported in eastern European countries (14.6%) [9] and in Brazil (10.3%) [20]. The high prevalence of physical abuse, neglect and exposure to domestic violence in the current study could be due to Iraq and Iraqis being exposed to different kinds of violence and traumatic events for decades, especially after the 2003 US invasion of Iraq, which had serious consequences on the behaviours of individuals and families [12, 22].

The proportion of emotional neglect was similar to what was reported in Brazil (19.7%) [20] and in Germany (19.1%) [3]. The literature shows that psychological maltreatment was the main form of child abuse in many countries [19, 20].

The results revealed that sexual abuse was not uncommon, with rates of sexual abuse (7.5%) higher than those reported in Lithuania (3.5%), in Romania (5.8%) [10] and in Brazil (1.4%) [19]. However, it was lower than what was reported in Germany (12.3%) [3] and at a similar rate to university students in Turkey (7.9%) [4].

The study showed that the prevalence of sexual abuse was higher among men than women, which is inconsistent with other studies [2, 4, 20]. This might be due to culture and generally insecure condition of Iraq; as sexual abuse is a very sensitive issue and is stigmatised, it may be that there was under-reporting of the prevalence of sexual abuse among women who may have been afraid to report such events, even though the questionnaire was anonymous and confidential. Furthermore, sexual abuse might occur during early childhood and individuals may be unable to remember such events, leading to recall bias.

The association between exposure to different forms of ACEs (neglect and abuse) with depression, and the association between a high ACE score and depression, is consistent with what has been reported in other studies [21, 23]. Traumatic events during childhood have prolonged effects on the personality and emotions of individuals, with many studies showing that the abnormality of the hypothalamic-pituitary-adrenal axis is a regular consequence of ACEs and might be the cause of psychopathological conditions in adults [24].

The results revealed no significant association between domestic violence and depression. The lack of association may be because male domination in relationships is an accepted norm, and therefore is not associated with depression. Or, given the continuous stressful conditions Iraqi families have experienced for a long time, it may be that other forms of violence exposure, such as to war, are more immediately important [22].

There was a difference between genders for the association between depression and exposure to certain forms of ACEs, as women were affected mainly by emotional neglect and mental illness in the household. Various studies have shown the association of women’s higher reporting of emotional abuse during childhood and worse health outcomes [21, 25, 26].

## Limitations

There were certain limitations to this study. First, it was difficult to provide a randomised sample of young adults (some young adults were students, others were working, while others were neither working nor studying). Furthermore, the current unstable situation of Iraq prevented visiting houses to conduct household to household surveys. Second, while we used forward translation procedures for the questionnaire, backward translation was not conducted. Third, adjusted regressions for the analyses were not done, so there might be underlying confounders.

## Conclusion

ACEs are not uncommon among young adults in Iraq and are associated with depression in adulthood. National programmes to support mental health rehabilitation might be necessary to reduce the effect of ACEs among Iraqis, especially for adolescents and young adults.

## Data Availability

The datasets used and/or analyzed during the current study available from the corresponding author on reasonable request.

## Abbreviations

ACEs: Adverse Childhood Experiences
DASS: Depression, Anxiety and Stress Scales
SPSS: Statistical Package for Social Science
US: United states of America
